# Animated PowerPoint Videos: An Underutilized Anatomy Educational Tool?

**DOI:** 10.1007/s40670-024-02007-x

**Published:** 2024-03-05

**Authors:** Eleni Patera, Munesh Pal Khamuani

**Affiliations:** 1 Section of Anatomy, St George’s University of London, London, UK; 2 Postgraduate Program in Medical Education, University of Nottingham, Queen’s Medical Centre, Nottingham, UK; 3 School of Medicine, Faculty of Health Sciences and Wellbeing, University of Sunderland, Sunderland, UK

**Keywords:** Anatomy, Anatomy education, Anatomy digital resource, E-Learning, Dental education, Dentistry, Drawing, PowerPoint

## Abstract

The subject of anatomy is an integral component of medical and dental education which are constantly evolving. Hence, educators continuously attempt to take advantage of technological advancements to create resources that will improve students’ higher cognitive skills. This article describes the creation of an e-learning resource in the form of an animated PowerPoint video that was designed based on evidence-based principles and educational theories to introduce the concept of tooth anatomy. Additionally, it outlines how this resource can be potentially integrated into a broader educational system as well as encourage anatomy and medical educators to use less complex technological equipment to create accessible educational resources.

## Introduction

In recent years, rapid technological advancements have resulted in the creation of plenty of teaching and learning educational tools in anatomy and medical education. Despite this, not all educational resources are freely available to medical students and students from allied health professions. With the advent of 3D technological educational resources, less attention has been given to educational resources that can be created with less complex technological equipment. Despite this, some articles discuss how YouTube [1–3], drawings [4–9], and PowerPoint [10, 11] can be used as educational tools.

In recent literature, there is limited evidence supporting the launch and use of YouTube videos as an anatomy educational resource that may support students in their anatomy learning [1–3, 12]. In a study, YouTube videos relevant to the anatomy learning objectives and teaching material were recommended to medical students to watch to supplement their learning [2]. Results showed that most students who used the content available on the institution’s YouTube channel thought it was a useful tool that assisted them while learning anatomy [2]. Despite this, evidence in the literature on the use of YouTube in medical education has not always been positive. A research report on the usefulness of YouTube in learning the anatomy of the heart assessed 294 heart anatomy videos that used different teaching resources ranging from plastic models, cadaveric material, lectures, illustrations, and animations to plastinated specimens. These videos were assessed in terms of their quality, content, and the anatomical accuracy of the information. Results from this study showed that 86.7% of the reviewed anatomy heart videos were uploaded by individuals while only 13.3% were uploaded by institutions [12]. Additionally, based on the scoring system that the authors used to objectively review these videos, only 25.9% of the videos achieved an overall pass [12]. Hence, this highlights the need for medical institutions to create their own reliable video resources that contain accurate anatomical information and can be easily accessed by students [12]. Evidence suggests that 78% of students access YouTube as a source of information when they encounter difficulty with their anatomy learning as it is easily accessible, free, easy to use, and not location dependent [13, 14]. Educators can upload their educational videos on the corresponding web-based learning managing system, such as Canvas they use at their institution; however, by uploading them on YouTube, they make the educational resource more accessible as it can be used by students outside their institution. Hence, for the aforementioned reason, the narrated PowerPoint video described in this monograph was uploaded on YouTube.

### Educational Problem and Rationale for Designing the Resource

Notably, there has been a reduction in the hours of anatomy teaching in medical and dental schools [15–22] and variability in terms of the teaching approaches adopted by medical and dental schools as not all of them can afford cadaveric dissection due to high cost and limited curricular time [23]. Even though limited, there is evidence in the literature that reports that anatomy in the undergraduate pre-clinical years is taught by non-medically or dentally qualified staff and this raises concerns in terms of whether such faculty are being trained sufficiently to be able to teach the clinical relevance of anatomy that may help students in their clinical medical or dental practice [24]. The knowledge of tooth anatomy serves as a basis for the management of pulpal and periradicular diseases along with restoring tooth function and aesthetics [25, 26]. Although there is limited evidence from the UK, wider educational literature does support the importance of anatomy education for clinical dental practice [27–29]. Empirical evidence on whether dental students can relate anatomy to their clinical practice better when taught by a qualified dentist is needed.

This article aims to discuss the potential use of an animated PowerPoint as an anatomy e-learning resource that is made available as a narrated video on YouTube. The animated PowerPoint video relies on a hand-drawn anatomy drawing of a single-rooted tooth which is explained by the narrator with key anatomical information summarized in bullet points. The educational video described in this article aims to help undergraduate preclinical dental students build a strong foundation of tooth anatomy from a clinical perspective. The rationale and need behind creating this e-learning resource to support dental students, drawing upon relevant literature and evidence-based principles, are also discussed. Lastly, this article briefly discusses how this e-learning resource can be integrated into the broader educational system.

## Description

In an attempt to evaluate how technology can enhance education, an e-learning resource, using PowerPoint and an anatomy drawing along with narrations and additional built-in functionalities within it, was developed. Eventually, this animated PowerPoint was published as an anatomy educational video on YouTube. Before the creation of this resource, YouTube, and other online webpages, such as Wikipedia, and Pocket Dentistry were reviewed. Most of the videos did not align with the cognitive load principles and none specifically addressed the following learning outcome that targets undergraduate pre-clinical dental students:

Learning Outcome: “Describe the anatomical features of the periodontal ligament and the gingiva, and their relationships of the individual teeth” [30]

There is a breadth of tooth anatomy videos currently available on online platforms and websites that can be accessed at any time. While these resources share similar types of information, they differ substantially from each other in terms of their design. However, most of these resources rely heavily on the use and combination of different multimedia elements such as images, text, sound, animations, and videos. The use of various multimedia elements all at once can distract the learner or lead them to experience cognitive overload; therefore, educators should use multimedia elements sparingly and thoughtfully [31, 32]. Yet, this raises concerns over the potential impact poorly designed online educational resources may have on students’ cognition and subsequently learning as well. Hence, there is a need for creating educational resources that are designed effectively by using evidence-based principles.

### Plan for Creation of the E-Learning Educational Resource

Spencer (2003) highlighted the importance for an instructor to map out a plan for their teaching sessions [33]. A similar approach was used for the development of the e-learning resource described in this article. Undergraduate first year dental students were the intended audience and the topic that the e-learning educational video aimed to cover was the basic anatomy of the tooth. The rationale behind choosing this topic and determining whether the topic is appropriate for first year dental students was that the anatomy of a tooth is one of the learning objectives of the anatomy dental curriculum as published by the Anatomical Society in 2020 [30]. As the aim was the creation of a multimedia e-learning resource to teach the basic anatomy of the tooth, a hand-drawn anatomy drawing of a single-rooted tooth, narration, and simple animations were used. In terms of how the anatomy drawing of a single-rooted tooth was chosen, it was ensured that the drawing was chosen from a clinical dentistry textbook. The reason why the drawing was not simply chosen from an anatomical atlas or textbook is that most anatomy textbooks illustrate teeth without the periodontal fibres being present. Hence, an illustration of a single-rooted tooth was re-created from “Newman and Carranza’s Clinical Periodontology” clinical dentistry textbook. Subsequently, the information that would have been included as part of the narrated script needed to be determined. “Netter’s Head and Neck Anatomy for Dentistry” textbook and the “Newman and Carranza’s Clinical Periodontology” clinical dentistry textbook were the books that were used to obtain accurate anatomical and clinically relevant information concerned with the basic anatomy of the tooth.

### Anatomy Drawing of a Single Rooted Tooth, Animations, and Narration

The anatomy drawing was drawn by the first author [EP] on A4 paper by using Staedtler Noris HB2 pencil and ARTEZA water-based ink color pens (ARTEZA, Inc). The drawing was drawn all at once, but it was gradually colored as the drawing was scanned at different stages and was incorporated into a Microsoft PowerPoint (version 16.55; Microsoft Office 2011) presentation to gradually demonstrate the different anatomical parts of a tooth and allow the viewer to focus on one topic at a time. Simple PowerPoint animations were used to gradually present the written information on each slide. Simple PowerPoint shapes were also used to introduce arrows that pointed out specific structures throughout the narration or other shapes that allowed the viewer to focus on specific narrated information. The recording option available on PowerPoint was used to record the narration for each PowerPoint slide. Eventually, the Open Broadcaster Software Studio platform (version 27.1.3; Open Broadcaster Software Studio, 2018) was used to combine the pre-recorded narrations with the PowerPoint’s animations which were manually added for the narrated information to match the written information eventually converting the PowerPoint into an mp4 video. The mp4 video lasted 7 minutes and 10 seconds and was uploaded under the second author’s YouTube account. Subtitles in English were autogenerated by YouTube after the video was uploaded on YouTube but were manually checked by the authors to ensure accuracy.

The resource was created using a personal laptop and Wi-Fi. No additional costs and equipment were required to create it. The educational video can be accessed using the following link: https://www.youtube.com/watch?v=XXQExhyfLDc

## Discussion

An online search of the anatomy dental educational videos on tooth anatomy available on YouTube was performed to identify if these videos were created based on evidence-based principles and whether they were inclusive or not. The online search revealed that most video resources on tooth anatomy did not align with the cognitive load theory. In contrast, the educational video described in this article was designed based on evidence-based principles. The two theories that informed the design of the e-learning resource described in this article are the multimedia learning principles and cognitive load theory [34, 35].

Multimedia learning simply means learning from words and pictures. Learning from words can be in the form of words written on a PowerPoint slide/on-screen text or spoken words while presenting material to the learner. In contrast, learning from pictures, often referred to as “pictorial learning”, is in the form of diagrams, animations, or other illustrations [31, 35]. Broadly, multimedia learning is a part of the “science of learning” (how people learn) which explains the information processing by the human brain via dual channels. Auditory or verbal channel processes verbal/auditory representation, whereas the visual channel processes pictorial or visual information. Nevertheless, both channels have a limited capacity to process information. However, one problem with multimedia learning that may arise is cognitive overload, where the processing demands for each of those channels exceed the cognitive capacity of the learner [36].

The principles of the multimedia and cognitive load theories were used to inform the design of the current educational video described in this article. These principles were applied to prevent the learners from quickly exceeding their cognitive capacity by managing the intrinsic cognitive load and optimizing the germane cognitive load. For that, the “simple-to-complex” approach was applied, by introducing the simple and basic material first and progressing towards the complex content. In addition, the extraneous cognitive load was reduced by utilizing both the auditory and visual modes of communication by incorporating the “modality principle” where pictures and labels were used to replace textual information with spoken explanation. In addition, the “redundancy principle” was incorporated by keeping the content relevant and specific to the learning outcome avoiding any unnecessary or irrelevant information [34, 37]. Furthermore, Mayer proposed “synchronizing” as one of the solutions to reducing cognitive overload [35]. This was ensured by using the additional animations and shapes feature in PowerPoint and presenting the corresponding visual and auditory information synchronously. Lastly, the educational resource described in this monograph had a duration of 7 minutes and 10 seconds. According to Bradbury (2016), the common consensus in the academic literature is that lectures should last between 10-15 minutes, as this is the attention span of most modern students [38]. Despite this, Bradbury emphasizes the need for studies with good study design that assess and measure attention and can therefore, provide more empirical evidence [38].

### Inclusivity and Accessibility Aspects of the Resource

This e-learning resource was designed based on the *Dyslexia Style Guide 2018: Creating Dyslexia Friendly Content* that was published by the British Dyslexia Association [39]. A single crème color background and black text written in the readable and dyslexia-friendly font style Calibri were used to ease written communication and to ensure adequate contrast between the background and the text. Upper case letters were avoided to facilitate written communication for individuals who have dyslexia. Furthermore, the video does not contain the red and green colors together, as this may disadvantage learners with visual impairments, such as red-green color blindness [40]. Subtitles corresponding to the spoken/vocal script were autogenerated by YouTube and were manually checked to ensure accuracy. The availability of captions on YouTube makes it accessible for learners with either hearing difficulties or those who are non-native speakers. Since the video is available online, learners who wish to revisit the video can do so many times. The video can also be downloaded and watched offline. Generally, the slide design, font, color, background, text, and layout in the video are aligned with those recommended for individuals with dyslexia [41, 42].

### How the E-Learning Resource Adds Value for Learners

#### Cost-Effective Valid Content

Firstly, the information provided in the video was not just taken from an anatomy textbook but from other clinical specialty textbooks, such as Periodontology and journal articles along with the authors’ clinical [MPK] and academic experience [EP, MPK]. Moreover, knowing the relevance of why the students are learning what they are learning (clinical relevance) can help to maintain their intrinsic motivation to study and learn as well [43]. Since the video is available on YouTube, there is no extra cost for the learners to pay excluding the broadband or internet data that they might already be paying for.

### How the E-Learning Resource Integrates into a Broader Educational System

Since there has been a reduction in anatomy teaching hours, this resource can help educators save time as they can embed it directly onto any virtual learning environment/learning management system (i.e., Moodle, Blackboard, BrightSpace, Canvas) for their students to watch in their own time. The e-learning resource can be used by all dental schools to educate their students. Moreover, it is evident from the literature that educational videos for dental students on YouTube are useful and carry high informational value [44]. Nevertheless, a study reported that 76.5% of the students gave high credibility scores to electronic resources recommended by faculty at their university. This suggests that e-learning can successfully be incorporated into the dental curriculum to help students apply their knowledge to clinical scenarios if reviewed by faculty [45] since students visiting non-peer-reviewed content on YouTube or any other online platform (particularly not recommended by a university) can come across conflicting information [46].

A study by Barry et al. [13] at Trinity College Dublin reported the reliance of medical students on web-based resources where YouTube was selected by 78% of students as their primary source of anatomy videos. These findings suggest that there is possible room for anatomy educators to integrate these videos as part of a blended learning curriculum or when implementing the flipped classroom approach. Nevertheless, as mentioned earlier, content review by using a scoring system before disseminating any videos to students is crucial. Institutions can also incorporate the e-learning resource as flipped classroom approach or recommend students to use it as an adjunct learning resource.

### Flipped Classroom Learning

Students can be encouraged to watch the video before the classroom session and the classroom time can be used to comprehend the 3D orientation and arrangement of the tooth layers in a real tooth thereby making effective use of technology before the classroom. A review by Kellesarian [47] highlights the importance of flipped classrooms in medical and dental education as it allows the students to take ownership of their cognitive and psychomotor skills with the facilitator mainly supporting the student learning process. A study at Harvard School of Dental Medicine used a model that combined active learning with technology, so the students use online resources to learn before attending the class and then use the classroom time for discussion. Students felt the exercise motivated them to participate in the class and helped them take ownership of their own learning [48].

### E-Learning Resource as an Adjunct to Primary Educational Resources

Nevertheless, this type of anatomy e-learning resource can be simply used by students as an adjunct to the primary educational resources their institution provides them with. Anatomy educators at dental institutions can create a series of educational videos addressing different learning objectives from the dental curriculum.

### Challenge Associated with Creating the E-Learning Resource

A challenge associated with creating this e-learning resource might be the use of hand drawing. Some may argue that not all anatomy educators exhibit artistic skills; therefore, they will be unable to recreate this resource or create a similar one. While this might be true to some extent, nowadays, there are various digital art software that facilitate the creation of digital drawings. However, such software may require specific equipment such as iPad devices and pens or require a monthly or annual subscription fee to use, which happened to be the reason behind why the drawing in the described resource was not digital and was hand-drawn instead.

### Limitations of E-Learning Resource

It is important to acknowledge the limitations associated with the e-learning resource. Since the resource requires an internet connection and a device to access the video, it may limit students who are in areas where there is no access to the internet or necessary equipment. Although YouTube does tell the views on how many people watched the video, it cannot evaluate whether learning has taken place. Also, even though the red and green colors in the described resource were not used as contrasting colors, it is important to acknowledge that these colors are often used by educators and within anatomy textbooks to illustrate blood vessels and lymphatics, respectively [49]. Another potential limitation of the resource could be the lack of an engaging activity or practice questions embedded within the video that can help the user assess their progress. One study aimed to investigate whether the incorporation of quizzes within pre-class videos that first year pharmacy students needed to watch prior to attending a lecture resulted in better memory retention, student performance, and amount of time spent on watching the pre-class videos [50]. The study showed that students who completed the quizzes that were embedded in the pre-class videos performed similarly for both the pre- and post-class quizzes to students that completed the quizzes after they watched the pre-class videos [50]. Despite this, students who completed the quizzes that were embedded in the pre-class videos were more likely to view these videos in contrast to students that watch the pre-class videos that did not contain any quiz questions in them [50]. Even though there was no statistically significant difference in terms of the amount of time students from the two groups spent on watching the pre-class videos, students who watched the pre-class videos that contained embedded quiz questions spent more time watching the pre-class videos; however, this could be justified by the fact that students needed more time as they needed to answer the embedded quiz questions within the pre-class videos [50]. Finally, a major limitation of the e-learning resource described in this monograph is that it has not been evaluated by dental students in terms of its effectiveness in facilitating students’ anatomy learning; therefore, there is a need for direct feedback from dental students in the future.

## Conclusion

Evidence-based principles informed the design of an e-learning resource which aimed to introduce the concept of the basic anatomy of the tooth to undergraduate pre-clinical dental students. Animated PowerPoint videos are digital multimedia learning resources that can be developed in a time- and cost-efficient manner. By using less complex technological equipment, medical educators can create animated PowerPoint videos to cover specific learning objectives. Animated PowerPoint videos can be used by students as an adjunct to their primary learning resources; despite this, further research to evaluate students’ perceptions and the effectiveness of such resources in helping students achieve specific learning outcomes is required.
